# A Novel Automatic Lung Nodule Classification Scheme using Fusion Ghost Convolution and Hybrid Normalization in Chest CTs

**DOI:** 10.2174/0115734056330120250310053454

**Published:** 2025-04-29

**Authors:** Yu Gu, Nan Wang, Jiaqi Liu, Lidong Yang, Baohua Zhang, Jing Wang, Xiaoqi Lu, Jianjun Li, Xin Liu, Siyuan Tang, Qun He

**Affiliations:** 1 Inner Mongolia Key Laboratory of Pattern Recognition and Intelligent Image Processing, School of Digital and Intelligent Industry, Inner Mongolia University of Science and Technology, Baotou 014010, China; 2 School of Automation and Electrical Engineering, Inner Mongolia University of Science and Technology, Baotou, 014010, China; 3 School of Information and Electronics, Beijing Institute of Technology, Beijing 100081, China; 4 College of Information Engineering, Inner Mongolia University of Technology, Hohhot, 010051, China; 5 School of Computer Science and Technology, Baotou Medical College, Inner Mongolia University of Science and Technology, Baotou 014040, China

**Keywords:** Classification of pulmonary nodules, Ghost convolution, Normalization, Visualization, Computed tomography, Lung Nodule Analysis 16

## Abstract

**Objective:**

To address the low efficiency of diagnosing pulmonary nodules using computed tomography (CT) images and the difficulty in obtaining the key signs of malignant pulmonary nodules, a ghost convolution residual network incorporating hybrid normalization (GCHN-net) is proposed.

**Methods:**

Firstly, a three-dimensional ghost convolution with a small kernel is embedded in the GCHN-net. Secondly, we designed a hybrid normalized-activation module (TMNAM) that can handle the rich and complex features of lung nodules in both the deep and shallow layers of the network, and incorporating two different normalization methods. This allows the network to comprehensively learn the intricate relationships underlying the intrinsic features of lung nodules and enhances its capacity to classify the properties of unknown nodules. Additionally, to enhance the accuracy and detail of the category activation map, GradCAM++ is integrated into the third layer of the GCHN-net. This integration enables the visualization of specific regions within three-dimensional lung nodules that the model focuses on during its predictions.

**Results:**

The accuracy of the GCHN-net on the Lung Nodule Analysis 16 (LUNA16) dataset was 90.22%, with an F1-score of 88.31% and a G-mean of 90.48%.

**Conclusion:**

Compared with existing methods, the proposed method can greatly improve the classification of pulmonary nodules and can effectively assist doctors in diagnosing patients with pulmonary nodules.

## 1 INTRODUCTION

According to the Global Cancer Epidemiology Database 2020, it is estimated that there are approximately 2.3 million new cases and 1.8 million deaths globally due to lung cancer [1, 2]. Moreover, male individuals have a higher probability of suffering from lung cancer compared to female individuals, possibly due to their preferences and physiological structural differences [3, 4]. Lung cancer is often mild in its early stages; however, around 70% of cases are diagnosed at an intermediate or advanced stage [5-7]. Therefore, early detection and diagnosis are of utmost importance for individuals with lung cancer [8-10]. Although CT scans can capture the heterogeneity of pulmonary nodules, experts’ evaluation of CT images can result in errors and inefficiencies [11-13]. Computer-Aided Diagnosis (CAD) can assist in diagnosing the nature of these nodules [14-16].

In recent years, the application of deep learning in the field of medicine has shown significant advancements in CAD of lung nodules [17, 18]. This has led to a significant improvement in the management of lung nodule-related diseases. One notable contribution in this area is the work of Usman *et al*. [19], who proposed a multi-encoder-based network (MEDS-Net) based on radiologists' workflow. The method utilizes three encoders to process different types of inputs and optimizes the learning process through a self-distillation mechanism, which effectively improves the accuracy and reduces false positives in lung nodule detection. Correspondingly, Mkindu *et al*. [20] proposed a lung nodule detection system that utilizes a Vision Transformer encoder with shifted windows and a region proposal network to extract deep features. The system optimizes hyperparameters using Gaussian Bayesian optimization. Furthermore, Bao *et al*. [21] improved the YOLOv8 model to enable multi-type testing of solid, mixed, ground-glass, and microscopic lung nodules. Building on the success of accurate detection, the field of lung nodule analysis has further expanded to include crucial aspects such as segmentation and classification. Gu *et al*. [22] proposed an improved convolutional neural network (CNN) model based on the U-Net architecture. The model introduces a ResNeXt module and an attention mechanism to enhance the learning of nodal features of different shapes and sizes. Meanwhile, Usman *et al*. [23] proposed a multi-encoder-based self-adaptive hard attention network (MESAHA-Net) that integrates CT slices, backward maximum intensity projection (MIP) images, and region of interest (ROI) masks to achieve accurate slice-by-slice 2D to 3D segmentation of lung nodules. Murugappan *et al*. [24] investigated the DeepLabV3+ model, optimized using different pre-trained networks, to improve the efficiency and accuracy of lung segmentation in chest CT images. The experiments included binary and quadruple classification tasks, with adjusted image sizes and hyperparameters, to provide an efficient automatic segmentation method for Coronavirus Disease 2019 (COVID-19) detection. Cao *et al*. [25] proposed a two-stage robust nodule detection CNN (TSCNN). The first stage, based on an improved U-Net segmentation network, proposed a new sampling strategy for training to achieve high recall without excessive false positives. In the second stage, a dual pooling structure was incorporated into three 3D-CNNs to reduce false positives. This detailed segmentation lays a solid foundation for more precise classification.

To improve the classification performance of lung nodules in CT scans, researchers have proposed various innovative approaches. Xie *et al*. [26] proposed a multi-view knowledge-based collaborative (MV-KBC) deep model, which decomposes a 3D nodule into nine fixed views and captures multiple features of the nodule using a knowledge-based collaboration sub-model. Xie *et al*. [27] considered multimodality and proposed an algorithm called Fuse-TSD, which fuses texture, shape, and depth model learning information. The algorithm extracts features using gray-level co-occurrence matrix texture descriptors, Fourier shape descriptors, and deep CNN and fuses the classification results using an Ad Boosted backpropagation neural network. Wang *et al*. [28] proposed a new automatic and accurate classification method for lung nodules based on the idea of constructing different architectures to extract and fuse multimodal features. In addition, some studies have improved the classification performance by optimizing the model structure and parameter settings. Zhao *et al*. [29] proposed a new agile CNN framework that combines the advantages of LeNet and AlexNet and improves the performance of lung nodule classification through optimized parameter settings. Lima *et al*. [30] successfully used the network to accurately classify lung nodules with diameters of 5-10 mm by adapting stochastic search and simulated annealing algorithms. Ali *et al*. [31] proposed a transferable texture CNN based on the energy layer. This layer extracts texture information from the convolutional layer, thereby reducing the number of learning parameters and consequently lowering memory requirements and computational complexity. Additionally, several research efforts have introduced specific methods or techniques aimed at addressing particular challenges in the field. For example, Jiang *et al*. [32] addressed the challenges of inadequate fine-grained representation and limited interpretability of existing methods by proposing context attention mechanisms and spatial attention mechanisms, respectively. Al-Shabi *et al*. [33] proposed a global-local network that effectively analyzes the network by extracting local features from residual blocks and global features from non-local blocks. This approach efficiently enhances the network's performance by combining these features. Li *et al*. [34] established a classification model for the benign and malignant nature of lung nodules by combining the Lung Image Database Consortium and Image Database Resource Initiative (LIDC-IDRI) database with the Lung Nodule Analysis Grand Challenge (LUNGx) database and using a quantitative representation of texture information in imaging histology.

The above networks have improved the performance of lung nodule classification through various aspects, but there is still room for improvement in terms of model validity. Deep neural networks have been shown to be more effective than shallow networks in extracting complex features of lung nodules [35-37]. Deep networks are capable of learning higher-level abstract features, which is crucial for recognizing subtle structural changes and patterns. However, deep networks often face problems of slow convergence and overfitting due to their large number of parameters [38-40]. The root cause of these issues is the high complexity of deep networks, which can lead to models getting stuck in local optima during training and being overly sensitive to noise and outliers in the training data.

In addition, 2D networks may not perform as well as 3D networks in capturing the spatial heterogeneity of lung nodules [41-43]. 2D networks typically extract information from a single slice, ignoring the spatial relationships between neighboring slices. In contrast, 3D networks can process multiple slices simultaneously, thus better capturing the 3D structure and spatial features of the nodule. However, 3D networks also introduce higher computational complexity and memory requirements, which pose a challenge in practical applications.

To address the limitations mentioned earlier, we propose a residual network that integrates ghost convolution and hybrid normalization to automatically diagnose lung nodules. We employ 3D ghost convolutions with suitable kernel sizes in the convolution layers of the Ghost Module Block (GMBlock) to reduce computational complexity, allowing the network to rapidly attain optimum performance and extract advanced information about pulmonary nodules. Furthermore, we analyze the advantages and drawbacks of two normalization methods at various points in the network and devise a TMNAM with corresponding activation functions to enhance the model's ability to adapt to small batch data.

Deep and shallow 3D residual networks each have their strengths and weaknesses when dealing with complex images [44-46]. In consideration of this, we create five variations of the GCHN-net with different depths and select the optimal network structure based on their performance on the validation set. Additionally, to enhance the network's capability to accurately identify the critical lesion locations within pulmonary nodules, we expand class activation mapping to three-dimensional structures and visualize the classification outcomes of the GCHN-net. This operation increases the clinical value of the GCHN-net network.

## 2 METHODS

### 2.1 Overall Structure of GCHN-net

An overview of the entire network structure can be seen in Fig. (**1**), where the pink, black, and yellow boxes represent the components of the Trunk, TMNAM, and GMBlock, respectively. To begin, a 32×32×32 CT image of a lung nodule was input into the network, and the nodule's center was identified using the label file. From the CT sequence, a 32×32×32 cube was selected and randomly padded to 36×36×36. It was then cropped and normalized back to 32×32×32 dimensions. The preprocessed CT image was passed through the trunk layer, which extracted the initial features at a lower level. The feature maps obtained from the trunk layer are downsampled in localized regions through four GMBlocks. Finally, average pooling and a fully connected classifier were employed to simplify the output of the convolutional layers and provide a classification of the lung nodule's benign or malignant nature. To ensure the reliability of the results, class activation mapping visualization was applied to the final convolution after the last layer of the third GMBlock. The values of n, p, m, and q were determined by the number of layers in the network.

### 2.2 GMBlock

The structure of the GMBlock is represented by the yellow dashed box in Fig. (**1**). The structure utilized a 3D ghost convolution to reduce the number of network parameters and computational complexity. The convolutional kernels employed in the GMBlock consisted of small sizes, either 1×1×1 or 3×3×3. These kernels, along with the inclusion of auxiliary channels, contributed to additional feature expression capabilities. To enhance the robustness of the network and enrich the representation of complex features in lung nodules, two types of hybrid normalized-activation modules were deployed separately in the Trunk and GMBlock. Specifically, Layer Normalization (LN) was used in the Trunk layer, while Batch Normalization (BN) was used in the GMBlock.

#### 2.2.1 Ghost Convolution

In the optimization process of deep networks, the addition of more layers often results in increased computational costs [47-49]. In order to enhance the performance of GCHN-net while reducing the memory cost, we employ a novel convolution technique [50, 51] and integrate it as a 3D embedding into the network. Fig. (**2**) illustrates the overall framework of the ghost convolution.

It is assumed that for 3D Ghost convolution, the input tensor 
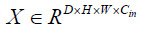
 , where D, H, and W are the depth, height, and width of the input feature map, respectively, and *C_in_* is the number of input channels. First, a standard small-scale 3D convolution is applied to generate a set of base feature maps, 
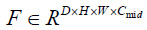
where 
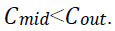
 Equation shown in (**1**):

**Table:** 

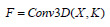	(1)

Where *K* represents the weights of the 3D convolutional kernel.

Next, a series of depthwise separable 3D convolutions is applied on the base feature maps *F* to generate additional feature maps 
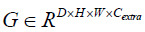
. The formula is shown below:

**Table:** 

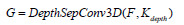	(2)

Where *K_depth_* represents the weights of the depthwise separable 3D convolution kernel.

Finally, the base feature map F and the generated feature maps *G* are concatenated to form the final output feature maps 
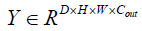
:

**Table:** 

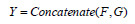	(3)

#### 2.2.2 TMNAM

During the network training process, the relationship between the adaptive distribution of the dataset and the features of lung nodule CT images can enhance the model's discriminative capacity [52-54]. Additionally, using a hybrid normalization technique in the network can automatically adjust the normalization parameters, effectively reducing the sensitivity of the inputs and thus improving the model's generalization ability [39, 55, 56]. Therefore, taking into account the normalization performance, in this experiment, a TMNAM was designed. The overall diagram of the TMNAM module is shown in Fig. (**1**).

Assuming the input sample is 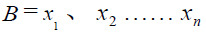, then after passing through the TMNAM module, Eq. (4) is obtained:

**Table:** 

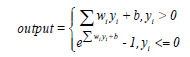	(4)

Where *y_i_* represents the result of normalizing; *w* and *b* represent the weight and bias of the network.

Since the trunk layer is located at the front end of the network, the feature map information at this stage is similar to the original image, and the batch size in this experiment is 2. However, it is improper to use BN [57-59], so LN [60, 61] is used at this stage. As the network gradually deepened after the backbone layer, the information in the image's visual content decreased. At this point, the influence of the small batch size was smaller than that of the image visual content, and therefore, it was better to normalize by taking the features of the same channel from different samples than by taking different channels from the same sample.

## 3 EXPERIMENT

### 3.1 Experimental Environment

The network model for this experiment was implemented using the PyTorch deep learning framework, and all experiments were conducted on a GeForce RTX 3090 GPU.

### 3.2 Dataset

The LUNA16 dataset [62-64] was employed in this experiment. It comprises 888 mhd format low-dose chest CT images obtained from the LIDC-IDRI dataset [65-67] after filtering out CT scans with a slice thickness exceeding 3mm and inconsistent slice spacing. The lung nodules selected for this study were based on the consensus of at least three radiologists. The LUNA16 dataset consisted of 10 compressed CT image files, each including an mhd file for metadata and raw files for the CT images.

For this experiment, Extensible Markup Language (XML) documents were used to categorize data with a malignancy grade of 3 as uncertain nodules and then were subsequently removed. Data with malignancy grades above 3 were classified as malignant nodules, while those below 3 were classified as benign nodules. The resulting dataset comprised 1008 nodules. In the training set, there were a total of 809 lung nodules, with 367 classified as malignant and 442 as benign. The validation set consisted of 103 nodules, of which 46 were malignant and 57 were benign. In the test set, there were 92 nodules, with 37 being malignant and 55 being benign. The distribution of each data set is shown in Table **1**.

### 3.3 Evaluation Criteria

The evaluation criteria used in this experiment were based on a confusion matrix. The results can be categorized into four cases: True Positive (TP), True Negative (TN), False Positive (FP), and False Negative (FN). The commonly used evaluation metrics in this experiment include Accuracy (ACC), Specificity (SPE), Sensitivity (SEN) and Precision (Pre). Additional metrics include the harmonic mean of Precision and Sensitivity, known as the F1-score, and the G-mean. The F1-score is used to assess the model's robustness, while the G-mean is used to evaluate the data imbalance. The formulas for these metrics are shown in Eqs. (**5**-**10**).

**Table:** 

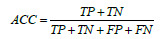	(5)

**Table:** 

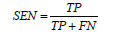	(6)

**Table:** 

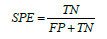	(7)

**Table:** 

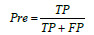	(8)

**Table:** 

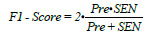	(9)

**Table:** 

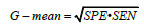	(10)

## 4 RESULTS AND DISCUSSION

### 4.1 Experimental Strategy

The experiment employed data augmentation techniques, such as random cropping, random horizontal flipping, and zeroing out, to enhance sample diversity and then reduce the likelihood of overfitting [68-70]. The optimizer used was AdamW, with first-order momentum and second-order momentum set to 0.999 and 0.99, respectively. The batch size, initial learning rate, and number of epochs were set to 2, 0.0002, and 200, respectively. In the experiment, the learning rate was kept constant for the first 130 epochs and then adjusted according to Eq. (11) for the remaining 70 epochs. The top three models with the highest ACC on the validation set were selected as the lung nodule classification models, which were used to test the proposed lung nodule classification model. Fig. (**3**) shows a comparison between the original images in LIDC-IDRI and the input images after cropping.

**Table:** 

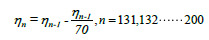	(11)

### 4.2 Skeleton Network Determination

The depth of the network can affect the model's performance [71-73]. Therefore, to find the optimal number of layers in the network, this study drew inspiration from the residual network [74-76] and constructed five basic frameworks, as shown in Fig. (**4**). This paper only shows the differences between the five networks in the GMBlock module, with ellipses representing the same Trunk, Avgpool, and FC as in Fig. (**1**). Compared with the GMBlock, the GMMBlock module omits ghost convolutions and TMNAM.

Fig. (**5**) shows the comparison results of different network architectures on three key metrics, namely, ACC, F1-score and G-mean. The figure indicates that the 50-layer network achieves an ACC of 87.38%, an F1-score of 85.71%, and a G-mean of 87.1% on the training set. The high ACC indicates that the model correctly classifies most of the samples, while the F1-score shows a good balance between Pre and SEN. The G-mean, which is particularly useful for unbalanced datasets, further confirms the robustness of the model across categories. Therefore, GCHN-net-50 was selected as the backbone network.

### 4.3 Contrast Experiments

In order to select the best module from the designed modules and thus maximize the effectiveness of the network, several contrast experiments were conducted, using the highest ACC on the validation set as a benchmark. All comparison experiments strictly followed the principle of controlled variables.

#### 4.3.1 Ghost *vs.* Traditional Convolution

Fig. (**6**) presents the comparative results of the two types of convolutions on the three metrics. The subscript “C” in the network denotes the use of ordinary convolutions in GCHN-net. When both types of convolutions were used in the network, the ACC obtained was the same for both. However, the F1-score and G-mean values of GCHN-net were slightly higher than those of the network using traditional convolutions, indicating that GCHN-net illustrates better recognition capabilities for pulmonary nodules. This may be because ghost convolution enhances the model's feature extraction capabilities and improves lung nodule identification by generating a more diverse set of feature maps. Therefore, ghost convolutions were selected in this study.

In this experiment, a comparison was made between the number of parameters and the computational workload of the two convolutions. Table **2** clearly demonstrates that for both k=1 and k=3, ghost convolution showed a smaller number of parameters and computational workload compared to ordinary convolution.

#### 4.3.2 Optimal Normalization Methods for Different TMNAM Layers

Four normalization methods were introduced in the TMNAM module of the trunk layer, and experiments were conducted with the Exponential Linear Unit (ELU). At this time, the TMNAM module in the GMBlock consists of BN and ELU. The comparative results are shown in Fig. (**7**). The networks denoted as B, W, and I in GCHN-net represent the backbone layers using BN, Weight Norm, and Instance Norm, respectively. As can be seen from the figure, when LN was used in this module, the ACC, F1-score, and G-mean values were 87.38%, 85.71%, and 87.1%, respectively, achieving the best performance in all metrics.

Experiments were conducted in the TMNAM module, specifically in the Trunk layer and GMBlock, by introducing four different activation function methods in conjunction with LN. At this point, the Trunk layer consists of LN and ELU, while the TMNAM module in the GMBlock consists of BN and ELU. The comparative results are shown in Fig. (**8**). The networks denoted as R, L, and G in GCHN-net represent the networks using Relu, LeakyRelu, and GELU activation functions, respectively. As can be observed from the figure, when ELU was selected in this module, all three metrics reached their optimal values.

To compare the performance of using LN in other positions of the network, LN was extended to other normalization positions in the network. The comparative results are shown in Fig. (**9**). The networks denoted as T, G, and D in GCHN-net represent the networks using LN in the trunk, GMBlock, and downsample layers, respectively. As can be seen from the figure, when LN with ELU was used in the trunk layer and BN with ELU was used in other positions, the model presented the best classification performance. This may be due to the fact that LN provides global stability and robustness at the Trunk layer, while BN maintains local feature consistency at the deeper layers. Combined with the nonlinear advantage of ELU, these factors improve the overall performance.

In summary, this experiment enriched the deep features of pulmonary nodules by using LN with ELU in the TMNAM of the trunk layer and BN with ELU in the TMNAM of the GMBlock layer. This ultimately enabled the GCHN-net to effectively recognize pulmonary nodule information.

#### 4.3.3 Optimal Optimizer and Learning Rate Strategy

This study conducted experiments by combining three optimizers, namely Adam, AdamW, and Stochastic Gradient Descent (SGD), with two learning rate optimization strategies. W and C represent the learning strategy mentioned in section 3.4.1 and the cosine learning strategy, respectively. As shown in Fig. (**10**), it can be observed that using the AdamW optimizer to optimize the network’s loss function along with the W learning strategy obtained the best results for pulmonary nodule classification. This may be due to the fact that the weight decay in AdamW effectively prevents overfitting and improves the model’s generalization ability. Additionally, the W learning strategy enables the model to converge faster and more stably, which further enhances the training effect and final performance of the model.

### 4.4 Ablation Studies

In order to validate the effectiveness of the selected modules in the contrast experiment, a series of ablation studies were carried out, using the highest test results as benchmarks.

The ablation studies were conducted on the network with the inclusion of the AdamW optimizer (base). The overall results of the ablation studies are summarized in Table **3**. It can be observed from Table **3** that introducing the TMNAM module and ghost convolution in the network can improve diagnostic accuracy. The proposed network in this study achieved the best classification results in terms of ACC, F1-score and G-mean, with values of 90.22%, 88.31%, and 90.48%, respectively. Compared to the base network, there were respective improvements of 5.44%, 7.76%, and 6.92%.

The sensitivity of GCHN-net was 91.89%. In terms of computational complexity and parameter, the reason GCHN-net was slightly larger than the base network is that it used 3x3x3 convolution kernels in the downsample layer of the network, which trades off the model parameter quantity for accuracy.

Tables **4**-**6** present the confusion matrix of the ablation models on the test set. It can be observed from the tables that the network designed in this study performed the best on the test set. It maintained the lowest false positive rate (5.36%) and false negative rate (13.89%). The true positive rate and true negative rate also reached maximum values of 86.11% and 94.64%, respectively.

Table **7** demonstrates the relationship between the number of parameters, the amount of computation, and the model confidence under different models. The percentages in parentheses in the table indicate the percentage increase relative to the Base network. From the table, it can be observed that as the model confidence increases, the number of parameters and the amount of computation of the model typically increases as well. However, when the TMNAM module was added to the Base network, the number of parameters and the amount of computation of the model decreased (by 0% and 0.23%, respectively), even though the confidence of the network increased by 3.59%. The reason for no change in the number of parameters may be that when the TMNAM module is added to the network, the relevant dimensions, such as the number of channels, are not changed, *i.e.*, the trainable parameters are not changed, and hence the number of parameters of the network remains the same. When ghost convolution was further added to the network, the confidence of the network continued to increase by 6.7%, but the number of parameters and computation of the model increased accordingly (by 28.98% and 8.55%, respectively). This is mainly due to the use of a 3×3 convolution kernel in the downsample layer of the network, which improves the confidence and ACC of the model. In the medical field, high classification accuracy is critical, as it directly determines diagnostic accuracy and is a stake in treatment outcomes and patient safety. Although the base + TMNAM + Ghost model configuration exhibits a significant increase in parameters and computational complexity, the corresponding improvement in confidence level justifies this tradeoff.

Figs. (**11** and **12**) depict the confidence maps of the correctly classified benign and malignant nodules and a visualized slice of a certain patient, respectively, generated by GCHN-net. The locations of the nodules are indicated by red boxes. From the size of the confidence score (P), it can be observed that the proposed network in this study is highly capable of distinguishing malignant nodules from benign ones. From a visualization perspective, the proposed network accurately locates the lesion regions (highlighted in red) of benign and malignant nodules.

#### 4.4.1 Visualization

Fig. (**13**) shows the learning process of the GCHN-net network after deploying the class activation mapping algorithm. The processed image was fed into GCHN-net, resulting in a final feature map that identified the nodule as malignant. During the backpropagation process, the weight of the feature map was obtained by calculating the gradient with respect to the pixels. By interpolating the colored mask image with the original image, the final visualization image was obtained using GradCAM++ [77-79].

Figs. (**14** and **15**) illustrate the visualization process of the correctly classified benign and malignant nodules in the test set using the proposed network, as well as the three-dimensional reconstruction of the original images. In the figures, (1) represents the sliced image after cropping. (2) represents the RGB image of the mask. Image (3) was obtained by interpolating images (2) and (1). The heatmap of the 32 slices is depicted in image (4). Image (5) visualizes the three-dimensional reconstruction of the lung nodules from the original images. The confidence scores for malignancy and benignity, as determined by the proposed network, were 0.9978 and 0.9660, respectively.

### 4.5 Performance Comparison Against State-of-the-art

To conquer the challenges of a small-scale medical image database, Zhao *et al*. proposed a new Agile CNN framework [29]. A study proposed a global-local network that enhances classification accuracy [33]. Another study, by Xie *et al*. constructing a knowledge-based collaborative (KBC) sub-model, proposed a multi-view knowledge-based collaborative (MV-KBC) deep model [26]. To address the issue of insufficient training data, Xie *et al*. proposed an algorithm that combines texture, shape, and other learning information [27]. Another study proposes a novel depth model for effectively classifying lung nodules by constructing a model process that utilizes oversampling algorithms, feature screening operations, and improved search algorithms. The authors introduce a TransUnet network in response to the challenges of accurately classifying micronodules using traditional methods, which are often subjective [28]. Lima *et al*. delved into algorithm tuning for a CNN, achieving high performance metrics through simulated annealing, thereby aiding in early and potentially life-saving diagnoses [30]. From Table **8**, it can be observed that the present study achieved the best results in terms of F1-score, G-mean, and computational complexity. Additionally, the SEN and ACC obtain the second-best results. Although the true positive rate and ACC are 0.15% and 0.02% lower than those of the network, respectively, the model size of this study's network was more than ten times smaller [32]. In conclusion, the network proposed in this study effectively captures key features of suspicious nodule lesions in a relatively lightweight manner.

## CONCLUSION

This paper proposed a deep residual network called GCHN-net that combines ghost convolution with hybrid normalization to accurately extract features of pulmonary nodule lesions using small kernel convolutions while reducing the number of network parameters. Based on gradient-weighted class activation mapping, ghost convolution can accurately locate key positions of pulmonary nodule lesions. Furthermore, this experiment discovered that hybrid normalization at appropriate positions and matching the activation function according to the normalization type could stimulate the network to adapt complex features, thereby improving the network's classification ability.

The final values of ACC, F1-score, and G-mean in this experiment were 90.22%, 88.31%, and 90.48%, respectively. The proposed model in this experiment not only demonstrated great classification performance on the LUNA16 dataset but also reduced the computational memory overhead of the model. Although GCHN-net demonstrated excellent classification performance and reduced computational memory overhead on the LUNA16 dataset, there are still some limitations in this study. Currently, the model is mainly based on CT scan data, and it is expected that the ability of the model to capture the characteristics of pulmonary nodular lesions will be further enhanced by integrating multi-modality data in the future. In addition, although we used Grad-CAM++ to improve the visualization and interpretation of the model, some aspects of the model still have some black-box characteristics, which, to some extent, affects its transparency in clinical applications. Future research will focus on the development of advanced multimodal fusion techniques and the introduction of interpretive AI methods to overcome these limitations, thereby further improving the diagnostic accuracy and clinical utility of the model.

## Figures and Tables

**Fig. (1):**
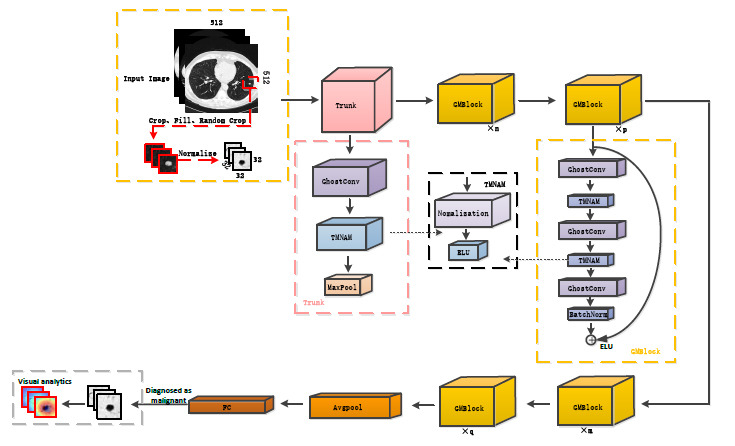
GCHN-net architecture.

**Fig. (2):**
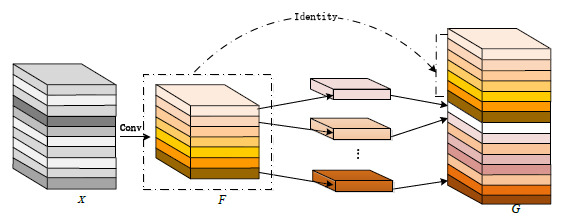
Ghost convolutional structure [50, 51].

**Fig. (3):**
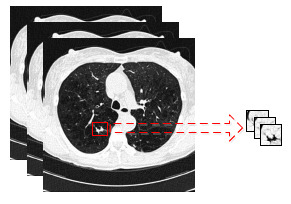
Original [65] *vs*. cropped images from LIDC-IDRI dataset.

**Fig. (4):**
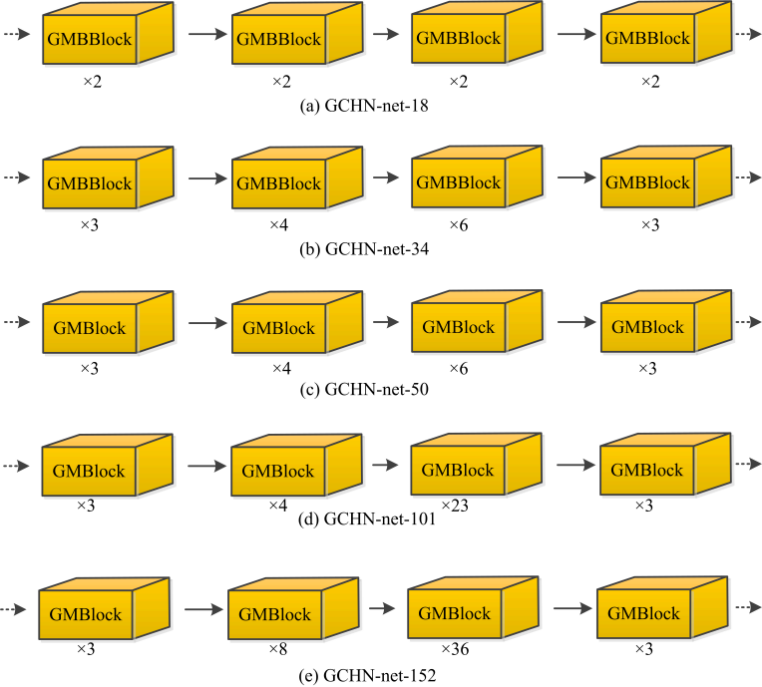
Structure diagram of each layer of GCHN-net.

**Fig. (5):**
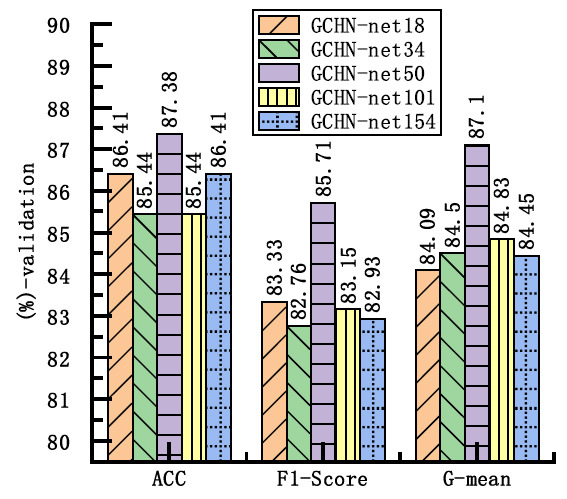
Performance comparison of GCHN-Net models across metrics.

**Fig. (6):**
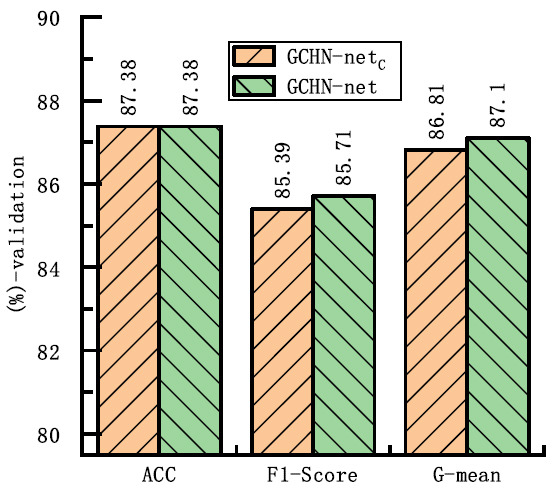
Convolutional comparison experiment.

**Fig. (7):**
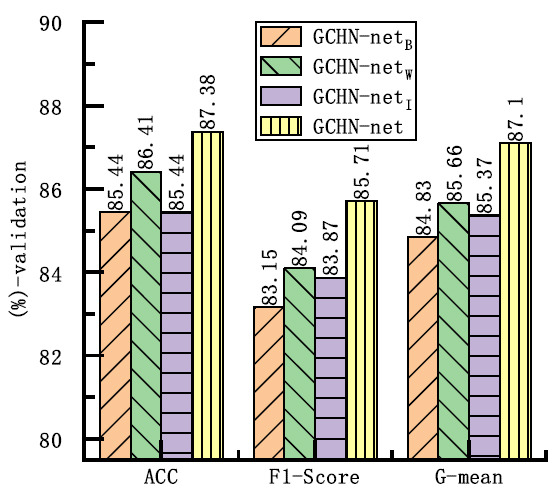
Comparative experiment on normalization methods.

**Fig. (8):**
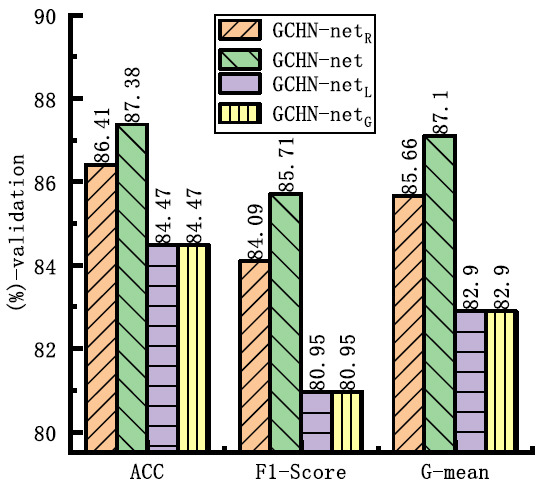
Comparative experiment of activation function.

**Fig. (9):**
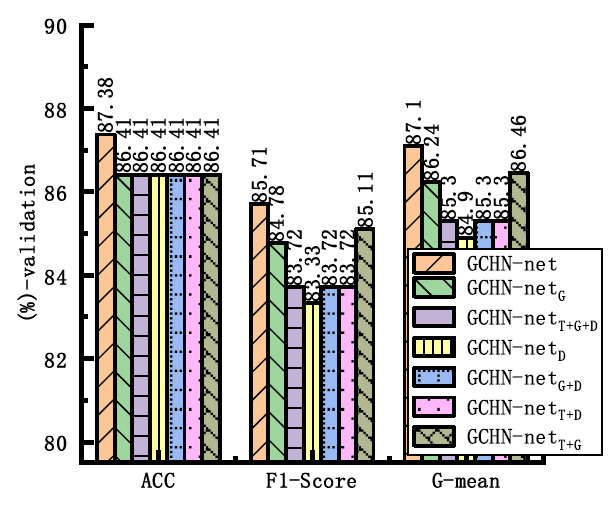
Position comparison experiment of layer normalization.

**Fig. (10):**
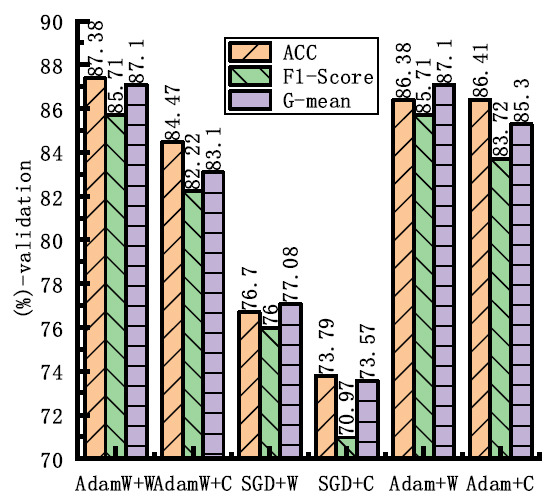
Comparative experiment on the combination of optimizer and learning rate optimization methods.

**Fig. (11):**
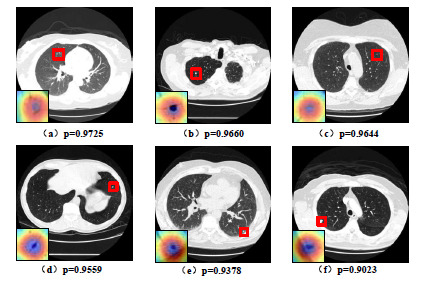
Partial correctly diagnosed benign pulmonary nodules in the LIDC-IDRI dataset corresponding to the original image, visualization heat map, and confidence level.

**Fig. (12):**
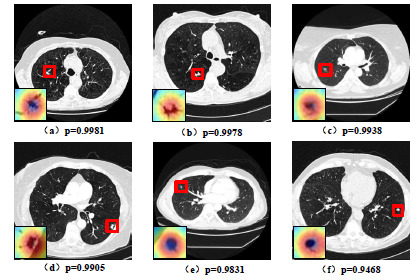
Partial correctly diagnosed malignant pulmonary nodules in the LIDC-IDRI dataset corresponding to the original image, visualization heat map, and confidence level.

**Fig. (13):**
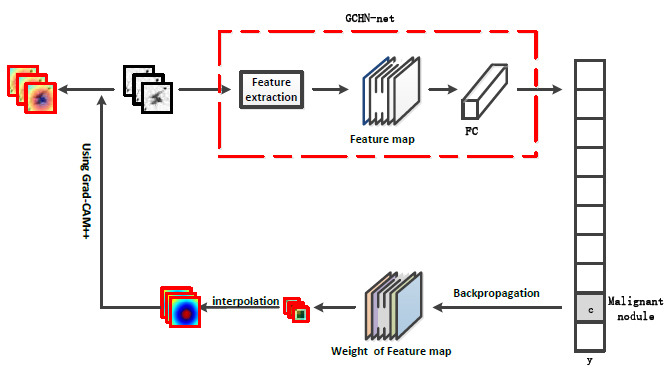
Visualization of GCHN-net-50 learning process.

**Fig. (14):**
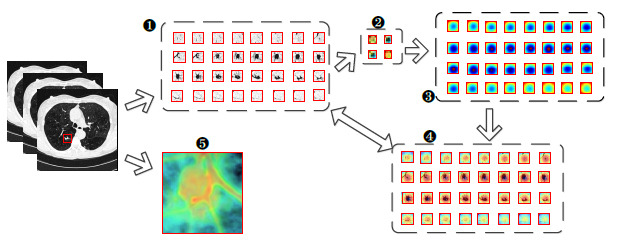
Visualization process and three-dimensional reconstruction of malignant nodules.

**Fig. (15):**
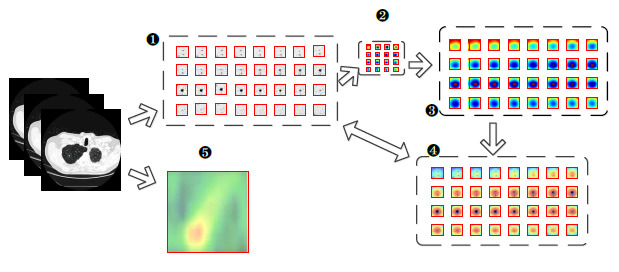
Visualization process and three-dimensional reconstruction of benign nodules.

**Table 1:** Training set, validation set and test set benign and malignant nodule allocation.

Dataset	Training Set		Validation Set		Test Set	
BN	MN	BN	MN	BN	MN
LUNA16	442	367	57	46	55	37

* BN indicates a benign nodule; MN indicates a malignant nodule.

**Table 2:** Comparison of params between Ghost convolution and ordinary convolution.

Convolution		Evaluation Indication	
GC	C	P (M)	F(Gmac)
1×1×1		44.82	4.36
3×3×3		80.81	6.76
	1×1×1	46.14	4.43
	3×3×3	118.13	9.23

* GC stands for Ghost Convolution, while C stands for Traditional Convolution. P stands for param, while F stands for FLOPs.

**Table 3:** The ablation studies of the pulmonary nodule classification network.

Method			Evaluation Metrics					
Base	TMNAM	Ghost	ACC	F1	G-mean	Params	FLOPs	SEN
√			84.78	80.55	83.56	**46.14**	4.44	78.38
√	√		86.96	83.78	86.40	46.14	**4.43**	81.08
√		√	90.22	86.96	88.39	59.51	4.81	89.19
√	√	√	**90.22**	**88.31**	**90.48**	59.51	4.82	**91.89**

* F1 and G_mean_ stand for F1-score, and G-mean, respectively. * **Bold** indicates the best result and a horizontal line with a different font represents the second best.

**Table 4:** Confusion matrix of base model.

Real Class		Predict Class	
P	N
P		31	5
N		5	51

**Table 5:** Confusion matrix of base + TMNAM model.

Real Class		Predict Class	
P	N
P		31	5
N		6	50

**Table 6:** Confusion matrix of base + TMNAM + Ghost model.

Real Class		Predict Class	
P	N
P		31	5
N		3	53

**Table 7:** Confidence versus model complexity.

Method				Evaluation Metrics		
Base	TMNAM	Ghost	Confidence	Params(M)	FLOPs (Gmac)	
√			79.48	46.14	4.44	
√	√		82.33(↑3.59%)	46.14(↑0%)	4.43(↓0.23%)	
√	√	√	84.83(↑6.7%)	59.51(↑28.98%)	4.82(↑8.55%)	

**Table 8:** Comparison with existing methods.

Method	F1-Score	G-mean	ACC	SPE	SEN	Params
Zhao *et al*. [29]	-	-	82.23	-	-	-
Al-Shabi *et al*. [33]	88.02	87.95	88.46	-	88.66	-
Xie *et al*. [26]	-	-	87.75	91.75	-	-
Xie *et al*. [27]	-	-	89.53	92.02	84.19	-
Jiang *et al*. [32]	-	-	**90.24**	88.94	**92.04**	678.69
Li *et al*. [34]	**-**	**-**	74.5	84.00	84.00	**-**
Wang *et al*. [28]	**-**	**-**	84.62	93.17	70.92	**-**
Lima *et al*. [30]	**-**	**-**	88.00	**94.00**	82.00	**-**
Ours	**88.31**	**90.48**	90.22	89.09	91.89	**59.51**

*Bold indicates the best result, and a horizontal line with a different font represents the second best.

## Data Availability

The data that support the findings of this study are openly available in LIDC-IDRI dataset at https://doi.org/10.1118/ 1.3528204, (reference number 65).

## LIST OF ABBREVIATIONS

ROI: Region of interest
MIP: Maximum intensity projection
CNN: Convolutional neural network
MEDS-Net: Multi-encoder-based network
CAD: Computer-Aided Diagnosis
LUNA16: Lung Nodule Analysis 16
CT: Computed tomography
